# Combined detection of inflammatory proteins is beneficial for diagnosing the papillary thyroid carcinoma and nodular goiter

**DOI:** 10.1002/imo2.14

**Published:** 2024-07-02

**Authors:** Yongqin Pan, Mingxi Xu, Tsz Hong Chong, Siping Xie, Kunsong Huang, Guanghao Wang, Yuhua Ma, Jinyi Li, Wah Yang

**Affiliations:** ^1^ Department of Thyroid Surgery The First Affiliated Hospital of Jinan University Guangzhou China; ^2^ Medical Record Room The First Affiliated Hospital of Jinan University Guangzhou China; ^3^ Department of Metabolic and Bariatric Surgery The First Affiliated Hospital of Jinan University Guangzhou China

**Keywords:** diagnosis, nodular goiter, papillary thyroid carcinoma, proximity extension assay

## Abstract

Fine‐needle aspiration cytology and imaging examinations are commonly used diagnostic tools for papillary thyroid carcinoma (PTC). However, these methods have limitations. Inflammatory proteins have the potential to serve as diagnostic and prognostic markers, as well as treatment targets. The expression profile and diagnosis effect of inflammatory proteins in PTC are not well understood. Here, 18 healthy volunteers (as healthy control), 12 patients with nodular goiter, and 34 patients with PTC were collected to analyze serum inflammatory proteins by proximity extension assay. Receiver operating characteristic curve analysis was used to evaluate the diagnostic potential of differential expression of proteins via the area under the curve (AUC) analysis. A total of 36 differentially expressed inflammatory proteins were found among PTC, nodular goiter, and healthy control. The combination diagnosis derived from the logistic regression analysis exhibited promising diagnostic capabilities in distinguishing nodular goiter from healthy control (AUC = 0.88), distinguishing PTC from healthy control (AUC = 0.89), and distinguishing PTC from nodular goiter (AUC = 0.87). Whereas the combination diagnosis derived from the least absolute shrinkage and selection operator (LASSO) exhibited promising diagnostic capabilities in distinguishing nodular goiter from healthy control (AUC = 0.92), distinguishing PTC from healthy control (AUC = 0.93), and distinguishing PTC from nodular goiter (AUC = 0.93). Overall, this study offers potential biomarkers for distinguishing between PTC and nodular goiter in clinical practice. The combination derived from the LASSO algorithm outperforms logistic regression.

## Highlights

- A total 36 differentially expressed inflammatory proteins were found among PTC, nodular goiter, and healthy control.
- TGF‐α + CXCL11 exhibited promising diagnostic capabilities in distinguishing nodular goiter from healthy control.
- TGF‐α + CXCL11 exhibited promising diagnostic capabilities in distinguishing PTC from healthy control.
- GDNF + CXCL5 + ARTN demonstrated optimal diagnostic effectiveness in distinguishing PTC from nodular goiter.
- The combination derived from the LASSO algorithm outperforms logistic regression, albeit with a higher number of proteins.

## Graphical abstract

A total 36 differentially expressed inflammatory proteins were found among PTC, nodular goiter, and healthy control. TGF‐α + CXCL11 exhibited promising diagnostic capabilities in distinguishing nodular goiter from healthy control. TGF‐α + CXCL11 also exhibited promising diagnostic capabilities in distinguishing PTC from healthy control. GDNF + CXCL5 + ARTN demonstrated optimal diagnostic effectiveness in distinguishing PTC from nodular goiter. The combination derived from the LASSO algorithm outperforms logistic regression, albeit with a higher number of proteins.

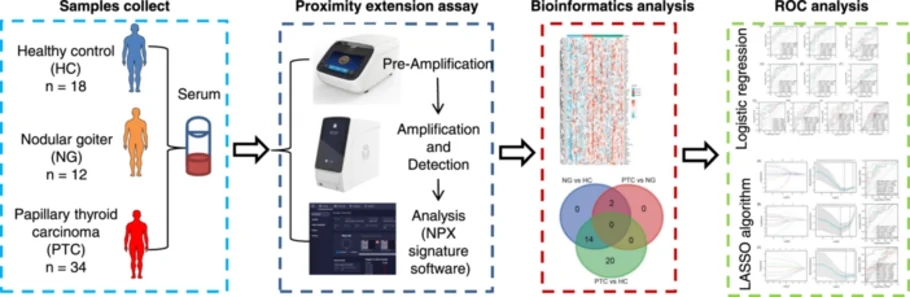

## 1 INTRODUCTION

Thyroid cancer, the most common endocrine malignancy, has seen a significant increase in its incidence rate since 2000, both in China and worldwide. However, the mortality rate has remained relatively stable during the same period [1]. In 2020, the age‐standardized incidence rates of thyroid cancer were 10.1/100,000 for men and 3/100,000 for women globally, with corresponding mortality rates of 0.5/100,000 and 0.3/100,000, respectively [2]. In 2019, the incidence and mortality rate of thyroid cancer were 2.05/100,000 and 0.39/100,000 [3]. Thyroid cancer can be categorized based on pathological types, including papillary thyroid carcinoma (PTC), follicular thyroid carcinoma (FTC), medullary thyroid carcinoma (MTC), and anaplastic thyroid cancer (ATC), with PTC accounting for approximately 80%−85% of all thyroid cancers. Fine‐needle aspiration cytology (FNAC) and imaging examinations are commonly used diagnostic tools for PTC in clinical practice. While imaging examinations can offer valuable preoperative guidance on thyroid invasion, they have a low positive rate for early tumor diagnosis. FNAC remains the gold standard for preoperative diagnosis of thyroid malignancy. However, it is an invasive procedure that carries the risk of severe complications and has a non‐diagnostic rate of about 10%−20% [4]. Therefore, there is still some uncertainty in diagnosing PTC, and there is a need to develop more convenient, safe, and accurate methods.

The immune system plays an important role in the development and progression of PTC. There is a positive correlation between chronic inflammation and the increased risk of thyroid cancer, which has attracted increasing attention as the main culprit mechanism of thyroid cancer occurrence [5]. Inflammatory proteins have been identified as potential diagnostic and prognostic markers as well as treatment targets [6, 7]. However, there remains some uncertainty surrounding the expression profile of these inflammatory proteins in thyroid cancer.

Proximity extension assay (PEA) is an innovative protein detection and quantification technology. PEA combines protein and antibody immune reaction techniques with oligonucleotide amplification techniques, utilizing unique antibody‐oligonucleotide protein binding and real‐time polymerase chain reaction (PCR) for quantitative measurement [8]. PEA exhibits high specificity, sensitivity, and detection throughput, making it an attractive option for detecting PTC biomarkers. This study aimed to evaluate the broad expression of inflammatory proteins in the serum of patients with PTC. By measuring 92 inflammation‐related proteins in the participants' serum, the study aimed to identify differential expression of inflammatory proteins in patients with PTC and to pinpoint potential diagnostic markers. This research has the potential to unveil new diagnostic biomarkers for PTC, offering valuable insights into the role of inflammation in thyroid cancer and potentially paving the way for improved diagnostic and therapeutic strategies.

## 2 RESULTS

### Characteristics of the patients

A total of 64 people were recruited for this study, including 29 males and 35 females. The average age of the participants was 37.80 ± 12.08. The baseline data for each group is presented in Table S1. The age, gender, free triiodothyronine, and free thyroxine had significant changes between nodular goiter and PTC.

### Differences in inflammatory protein among healthy control, nodular goiter, and PTC

**Figure 1:**
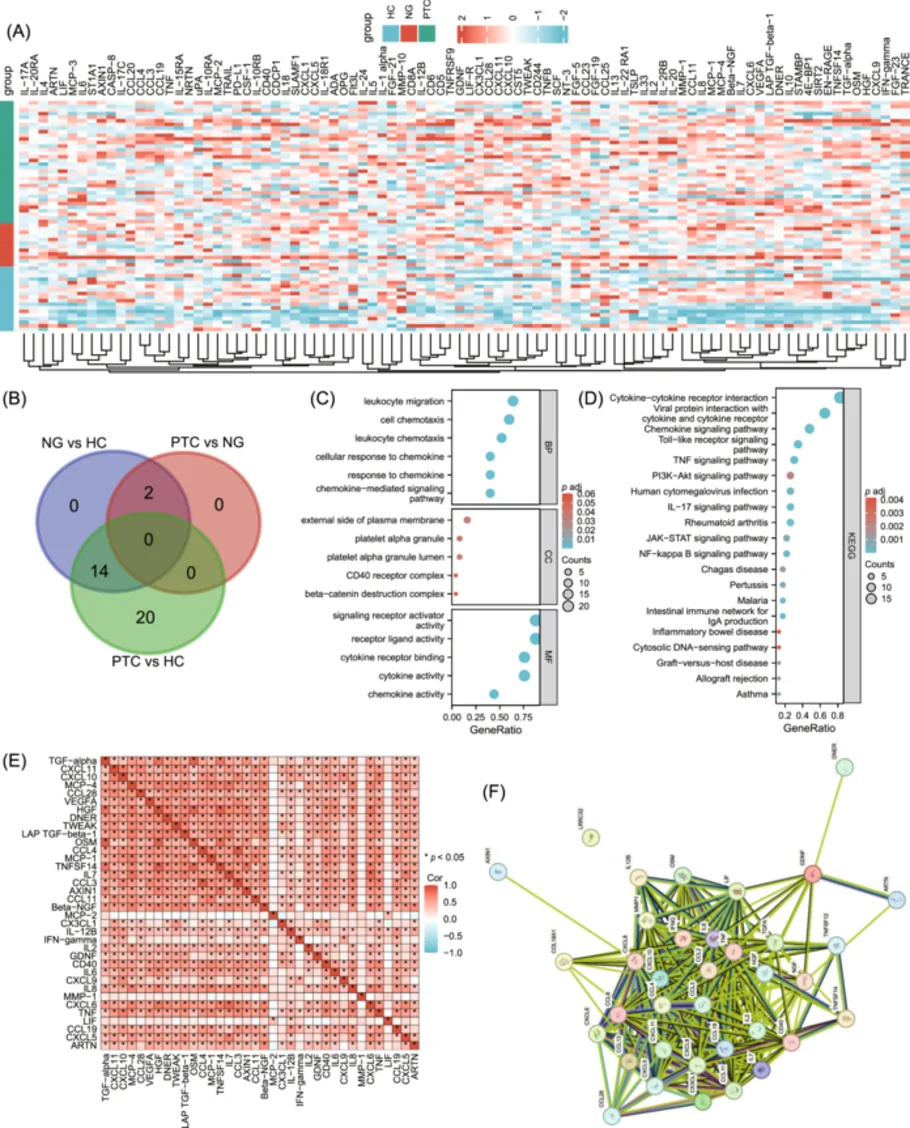
Function, regulatory signal pathways, and related analysis of inflammatory proteins after proximity extension assay (PEA) were analyzed. (A) Heatmap revealed differences in inflammatory protein levels between healthy control (HC), nodular goiter (NG), and papillary thyroid carcinoma (PTC). (B) The Venn diagram illustrates the intersection of the three groups. (C) Functional and signaling pathway enrichment analysis was conducted on these 36 proteins. (D) Signaling pathway enrichment analysis was conducted on these 36 proteins. (E) Correlation analysis focusing on the 36 genes. (F) Protein network analysis focusing on the 36 genes.

The expression of inflammatory proteins in healthy control, nodular goiter, and PTC samples was represented using a heatmap (Figure 1A). A comparison revealed 16 differentially expressed inflammatory proteins between healthy control and nodular goiter, 34 differentially expressed inflammatory proteins between healthy control and PTC, and 2 differentially expressed inflammatory proteins between nodular goiter and PTC. The Venn diagram in Figure 1B illustrates the intersection of the three groups, revealing 36 differentially expressed inflammatory proteins. Subsequently, functional and signaling pathway enrichment analysis was conducted on these 36 proteins, as depicted in Figure 1C,D. The analysis indicated a strong association between the differentially expressed inflammatory proteins and processes such as leukocyte migration, cell chemotaxis, receptor–ligand activity, signaling receptor activator activity, cytokine–cytokine receptor interaction, viral protein interaction with cytokine and cytokine receptor, as well as chemokine, NF‐κB, JAK–STAT, and PI3K–Akt signaling pathways. Furthermore, correlation and protein network analysis focusing on the 36 genes is displayed in Figure 1E,F. The outcomes highlight that most of these proteins belong to the CXCL family and interleukin class, exhibiting robust correlations and interconnections.

### The diagnostic accuracy of differences in inflammatory proteins was investigated to diagnose nodular goiter from healthy control

**Figure 2:**
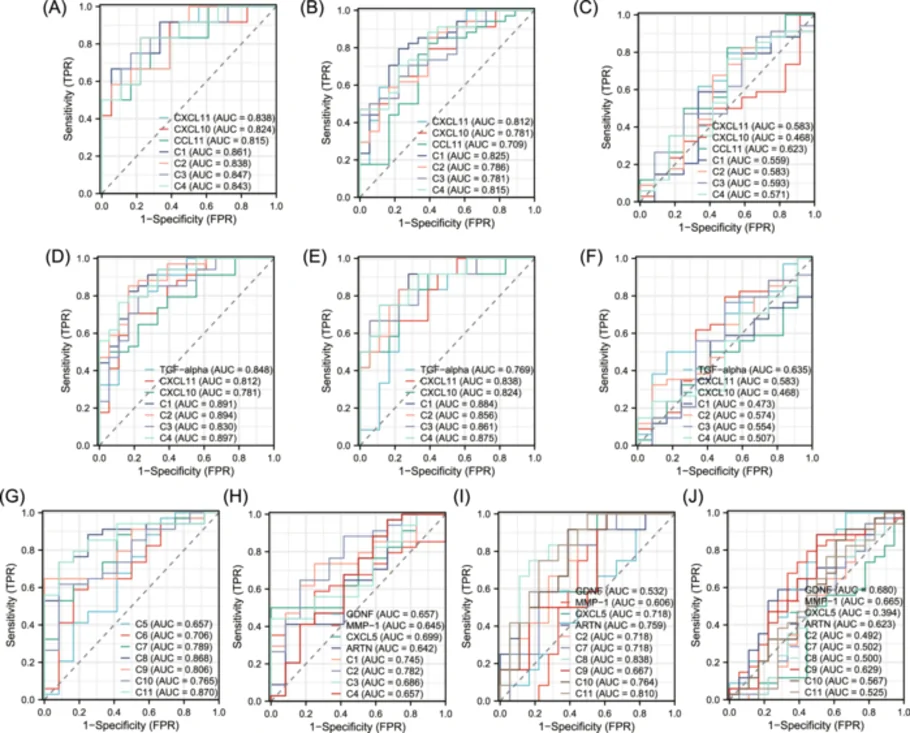
The best diagnostic efficacy in distinguishing between healthy control, nodular goiter, and papillary thyroid carcinoma (PTC) were analyzed via the receiver operating characteristic (ROC) curve. (A–C) ROC curve was used to determine the diagnostic performance of CXCL11, CXCL10, and CCL11 for nodular goiter from healthy control (A), for PTC from healthy control (B), and for PTC from nodular goiter (C); C1: CXCL11 + CXCL10; C2: CXCL11 + CCL11; C3: CXCL10 + CCL11; C4: CXCL11 + CXCL10 + CCL11. (D–F) ROC curve was used to determine the diagnostic performance of TGF‐α, CXCL11, and CXCL10 for PTC from healthy control (D), for PTC from healthy control (E), and for PTC from nodular goiter (F); C1: TGF‐α + CXCL11; C2: TGF‐α + CXCL10; C3: CXCL11 + CXCL10; C4: TGF‐α + CXCL11 + CXCL10. (G–J) ROC curve was used to determine the diagnostic performance of GDNF, MMP‐1, CXCL5, and ARTN for PTC from nodular goiter (G, H), for PTC from healthy control (I), and for PTC from nodular goiter (J); C1: GDNF + MMP‐1; C2: GDNF + CXCL5; C3: GDNF + ARTN; C4: MMP‐1 + CXCL5; C5: MMP‐1 + ARTN; C6: CXCL5 + ARTN; C7: GDNF + MMP‐1 + CXCL5; C8: GDNF + MMP‐1 + ARTN; C9: GDNF + CXCL5 + ARTN; C10: MMP‐1 + CXCL5 + ARTN; C11: GDNF + MMP‐1 + CXCL5 + ARTN.

The diagnostic efficiency of 36 different proteins in distinguishing nodular goiter from healthy control was evaluated using ROC curve analysis. The results indicated that CXCL11, CXCL10, and CCL11 showed the best individual diagnostic performance (Table S2). When combined with three proteins for diagnosis, the combination of CXCL11 + CXCL10 (C1) showed the highest diagnostic efficacy in distinguishing nodular goiter from healthy control (Figure 2A and Table 1), while the combination of CXCL11 + CXCL10 (C1) also exhibited the highest diagnostic efficacy in distinguishing PTC from healthy control (Figure 2B and Table S3). Combined with three proteins for diagnosis, CCL11 exhibited the best diagnostic efficacy in distinguishing PTC from nodular goiter. However, the diagnostic performance combined with CXCL11, CXCL10, and CCL11 was poor (Figure 2C and Table S4). These results indicate that CXCL11 + CXCL10 is a potential diagnostic biomarker in distinguishing nodular goiter from healthy control (area under the curve [AUC] = 0.86) and distinguishing PTC from healthy control (AUC = 0.83).

**Table 1:** The diagnostic accuracy of CXCL11, CXCL10, and CCL11 in distinguishing nodular goiter from healthy control.

Variables	Cut‐off value	Area under the curve	Confidence interval	Sensitivity	Specificity
CXCL11	>7.1794	0.84	0.690–0.986	0.67	0.94
CXCL10	>7.3432	0.82	0.661–0.987	0.83	0.78
CCL11	>6.9523	0.82	0.656–0.974	0.50	1.00
C1	>11.133	0.86	0.722–1.000	0.67	0.94
C2	>10.482	0.84	0.693–0.983	0.58	0.94
C3	>12.688	0.85	0.699–0.996	0.75	0.83
C4	>24.636	0.84	0.696–0.989	0.83	0.78

*Note*: C1: CXCL11 + CXCL10; C2: CXCL11 + CCL11; C3: CXCL10 + CCL11; C4: CXCL11 + CXCL10 + CCL11.

### Inflammatory biomarkers of different proteins were investigated for diagnosing PTC from the healthy control

The diagnostic efficiency of 36 different proteins in distinguishing PTC from healthy control was evaluated using ROC curve analysis. The results indicated that TGF‐α, CXCL11, and CXCL10 showed the best individual diagnostic performance (Table S2). When combined with three proteins for diagnosis, the combination of TGF‐α + CXCL11 (C1), TGF‐α + CXCL11 (C2), and TGF‐α + CXCL11 + CXCL10 (C4) showed the highest diagnostic efficacy in distinguishing PTC from healthy control (Figure 2D and Table 2). In addition, there was similar diagnostic efficacy among the three type combinations. The combined diagnosis of TGF‐α, CXCL11, and CXCL10 was further evaluated in distinguishing nodular goiter from healthy control and the combination of TGF‐α + CXCL11 (C1) and TGF‐α + CXCL11 + CXCL10 (C4) exhibited the highest diagnostic efficacy (Figure 2E and Table S5). However, the diagnostic performance of the combined with TGF‐α, CXCL11, and CXCL10 was poor when distinguishing PTC from nodular goiter (Figure 2F and Table S6). These results indicate that TGF‐α + CXCL11 is a potential diagnostic biomarker in distinguishing nodular goiter from healthy control (AUC = 0.88) and distinguishing PTC from healthy control (AUC = 0.89).

**Table 2:** The diagnostic accuracy of TGF‐α, CXCL11, and CXCL10 in distinguishing papillary thyroid carcinoma from healthy control.

Variables	Cut‐off value	Area under the curve	Confidence interval	Sensitivity	Specificity
TGF‐α	>2.856	0.85	0.731–0.965	0.91	0.67
CXCL11	>6.8231	0.81	0.686–0.938	0.71	0.83
CXCL10	>7.6828	0.78	0.656–0.906	0.47	1.00
C1	>8.6035	0.89	0.798–0.983	0.82	0.83
C2	>9.0794	0.89	0.804–0.983	0.85	0.83
C3	>10.651	0.83	0.711–0.949	0.85	0.72
C4	>10.125	0.90	0.814–0.981	0.76	0.89

*Note*: C1: TGF‐α + CXCL11; C2: TGF‐α + CXCL10; C3: CXCL11 + CXCL10; C4: TGF‐α + CXCL11 + CXCL10.

### Inflammatory biomarkers of different proteins were investigated for diagnosing PTC from nodular goiter

The diagnostic efficiency of 36 different proteins in distinguishing PTC from nodular goiter was evaluated using ROC curve analysis. The results indicated that GDNF, MMP‐1, CXCL5, and ARTN showed the best individual diagnostic performance (Table S2). When combined three proteins for diagnosis, the combination of GDNF + CXCL5 + ARTN (C8) and GDNF + MMP‐1 + CXCL5 + ARTN (C11) showed the highest diagnostic efficacy in distinguishing PTC from nodular goiter (Figure 2G,H and Table 3). The combined diagnosis of GDNF, MMP‐1, CXCL5, and ARTN was further evaluated in distinguishing nodular goiter from healthy control and the combination of GDNF + CXCL5 + ARTN (C8) exhibited the highest diagnostic efficacy (Figure 2I and Table S7). However, the diagnostic performance of the combined with GDNF, MMP‐1, CXCL5, and ARTN was poor when distinguishing PTC from healthy control (Figure 2J and Table S8). Overall, these results indicate that GDNF + CXCL5 + ARTN serves as a potential diagnostic biomarker in distinguishing PTC from nodular goiter (AUC = 0.87) and distinguishing nodular goiter from healthy control (AUC = 0.84).

**Table 3:** The diagnostic accuracy of GDNF, MMP‐1, CXCL5, and ARTN in distinguishing papillary thyroid carcinoma from nodular goiters.

Variables	Cut‐off value	Area under the curve	Confidence interval	Sensitivity	Specificity
GDNF	>0.5934	0.66	0.453–0.861	0.97	0.33
MMP‐1	>14.496	0.65	0.484–0.805	0.35	1.00
CXCL5	<10.888	0.70	0.540–0.858	0.50	1.00
ARTN	<−1.6589	0.64	0.448–0.836	0.41	0.92
C1	<10.001	0.75	0.592–0.898	0.74	0.75
C2	<9.8872	0.78	0.627–0.936	0.65	0.83
C3	<5.8099	0.69	0.522–0.851	0.44	1.00
C4	>0.10681	0.66	0.453–0.861	0.97	0.33
C5	>0.11341	0.66	0.453–0.861	0.97	0.33
C6	>18.149	0.71	0.526–0.885	0.59	0.83
C7	>−5.5887	0.79	0.643–0.936	0.88	0.58
C8	>−3.2765	0.87	0.759–0.976	0.74	0.92
C9	>4.3898	0.81	0.679–0.934	0.65	1.00
C10	<2.2831	0.77	0.614–0.916	0.62	0.92
C11	>−1.6956	0.87	0.764–0.976	0.74	0.92

*Note*: C1: GDNF + MMP‐1; C2: GDNF + CXCL5; C3: GDNF + ARTN; C4: MMP‐1 + CXCL5; C5: MMP‐1 + ARTN; C6: CXCL5 + ARTN; C7: GDNF + MMP‐1 + CXCL5; C8: GDNF + MMP‐1 + ARTN; C9: GDNF + CXCL5 + ARTN; C10: MMP‐1 + CXCL5 + ARTN; C11: GDNF + MMP‐1 + CXCL5 + ARTN.

### A diagnostic marker for thyroid cancer was developed using the least absolute shrinkage and selection operator (LASSO) algorithm

**Figure 3:**
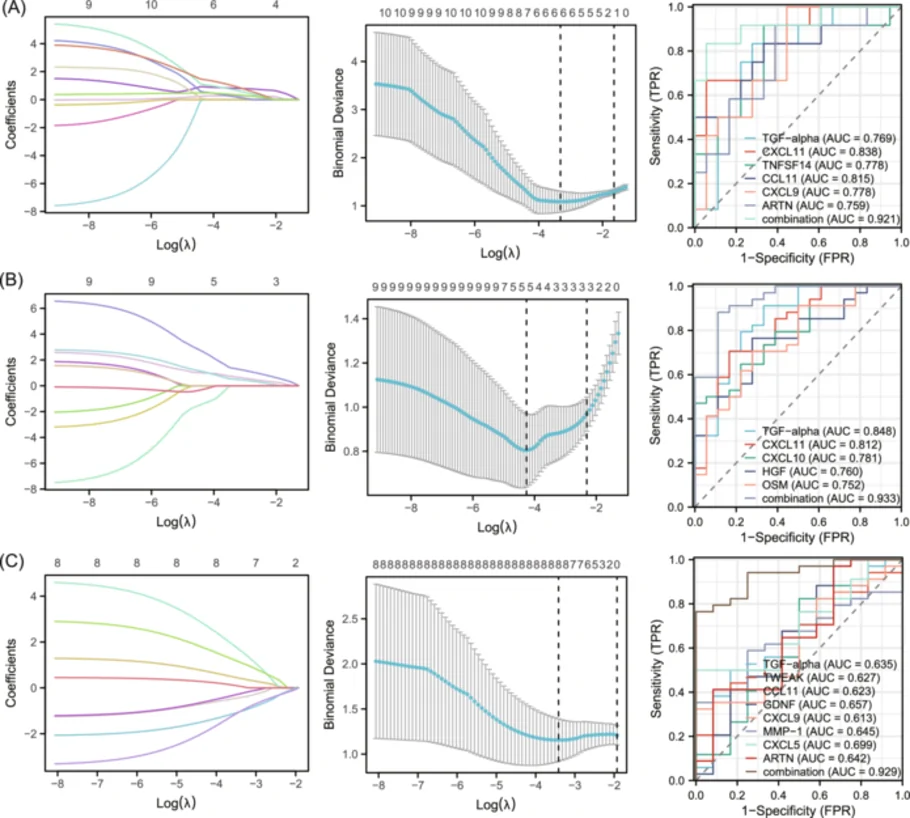
Diagnostic marker for healthy control, nodular goiters, and papillary thyroid carcinoma (PTC) was developed using the Least absolute shrinkage and selection operator (LASSO) algorithm. (A) LASSO coefficient curve, LASSO coefficient screening, and ROC curve for diagnosis of the nodular goiter from healthy control. (B) LASSO coefficient curve, LASSO coefficient screening, and ROC curve for diagnosis of the PTC from healthy control. (C) LASSO coefficient curve, LASSO coefficient screening, and ROC curve for diagnosis of the PTC from nodular goiter.

For diagnosis of the nodular goiter from the healthy control, LASSO regression was performed and identified six biomarkers for diagnosis of the nodular goiter from the healthy control (Figure 3A). For diagnosis of the PTC from healthy control, LASSO regression was performed and identified five biomarkers (Figure 3B). For diagnosis of the PTC from nodular goiter, LASSO regression was performed and identified eight biomarkers (Figure 3C). Combining those proteins for diagnosis after LASSO regression exhibited the best diagnostic efficacy (Table 4).

**Table 4:** The diagnostic accuracy in distinguishing healthy control, nodular goiters, and papillary thyroid carcinoma (PTC) by least absolute shrinkage and selection operator algorithm.

Variables	Cut‐off value	Area under the curve	Confidence interval	Sensitivity	Specificity
C1	>0.15	0.92	0.812–1.000	0.83	0.94
C2	>0.39	0.93	0.864–1.000	0.88	0.89
C3	>1.15	0.93	0.857–1.000	0.76	1.00

*Note*: C1 (nodular goiters vs. healthy control): TGF‐α, CXCL11, TNFSF14, CCL11, CXCL9, and ARTN combination; C2 (PTC vs. healthy control): TGF‐α, CXCL11, CXCL10, HGF, and OSM combination; C3 (PTC vs. nodular goiters): TGF‐α, TWEAK, CCL11, GDNF, CXCL9, MMP‐1, CXCL5, and ARTN combination.

### Analysis of risk factors for nodular goiter and PTC occurrence

The differential proteins, as well as age and gender, were included in a binary logistic regression analysis model. After controlling for confounding factors such as age and gender, compared to the healthy control group, seven proteins including TNFSF14, CCL11, ARTN, VEGFA, TGF‐α, CXCL11, and CXCL10 were found to be risk factors for nodular goiter occurrence (Table 5); furthermore, 28 proteins including TGF‐α, CXCL11, CCL4, MCP4, VEGFA, DNER, HGF, CXCL10, CCL28, TWEAK, MCP‐1, OSM, TNFSF14, CCL3, AXIN1, LAP‐TGF‐β1, MCP2, IL7, CCL11, β‐NGF, IL8, IL2, GDNF, IL6, LIF, IFN‐γ, CX3CL1, CXCL6 were found to be risk factors for PTC occurrence (Table 6) when compared to the healthy control group. Additionally, six proteins including TNFSF14, CCL11, VEGFA, TGF‐α, CXCL11, and CXCL10 were found to be common risk factors for both nodular goiter and PTC occurrence when compared to the healthy control group.

**Table 5:** Risk factor affecting the occurrence of nodular goiter was presented.

Characteristics	Total (*N*)	HR (95% CI)	*p* Value
TNFSF14	30	13.39 (1.40–128.47)	0.025
CCL11	30	15.26 (1.40–166.12)	0.025
ARTN	30	28.44 (1.25–647.35)	0.036
VEGFA	30	11.49 (1.17–112.82)	0.036
TGF‐α	30	8.21 (1.13–59.54)	0.037
CXCL11	30	4.93 (1.04–23.35)	0.044
CXCL10	30	8.22 (1.01–66.73)	0.049

*Note*: The binary logistics regression analysis model was used to assess the influence of 36 differential proteins on the occurrence of nodular goiter. Confounding factors of age and gender were controlled. Only proteins with significant effects are presented in the results.

**Table 6:** Risk factor affecting the occurrence of papillary thyroid carcinoma (PTC) was presented.

Characteristics	Total (*N*)	HR (95% CI)	*p* Value
TGF‐α	52	10.421 (2.547–42.632)	0.001
CXCL11	52	7.271 (1.905–27.752)	0.004
CCL4	52	4.786 (1.612−14.210)	0.005
MCP4	52	4.420 (1.552–12.589)	0.005
VEGFA	52	4.914 (1.591–15.178)	0.006
DNER	52	31.866 (2.730−371.887)	0.006
HGF	52	6.943 (1.745–27.629)	0.006
CXCL10	52	6.755 (1.728–26.403)	0.006
CCL28	52	5.770 (1.634–20.371)	0.006
TWEAK	52	12.142 (1.946–75.758)	0.008
MCP_1	52	9.067 (1.794−45.818)	0.008
OSM	52	2.817 (1.307–6.070)	0.008
TNFSF14	52	3.347 (1.354−8.272)	0.009
CCL3	52	4.162 (1.403−12.349)	0.01
AXIN1	52	11.459 (1.706−76.986)	0.012
LAP_TGFβ1	52	3.864 (1.328−11.238)	0.013
MCP2	52	2.334 (1.190−4.578)	0.014
IL7	52	3.588 (1.262−10.204)	0.017
CCL11	52	6.902 (1.391−34.252)	0.018
β‐NGF	52	155.360 (1.984−12,166.350)	0.023
IL8	52	3.700 (1.188−11.523)	0.024
IL2	52	7.166 (1.277−40.217)	0.025
GDNF	52	6.565 (1.205−35.761)	0.03
IL6	52	3.001 (1.074−8.389)	0.036
LIF	52	11.879 (1.171−120.537)	0.036
IFN‐γ	52	2.880 (1.057−7.844)	0.039
CX3CL1	52	7.101 (1.086−46.424)	0.041
CXCL6	52	2.437 (1.007−5.899)	0.048

*Note*: The binary logistics regression analysis model was used to assess the influence of 36 differential proteins on the occurrence of PTC. Confounding factors of age and gender were controlled. Only proteins with significant effects are presented in the results.

### The expression of main differences in inflammatory protein

**Figure 4:**
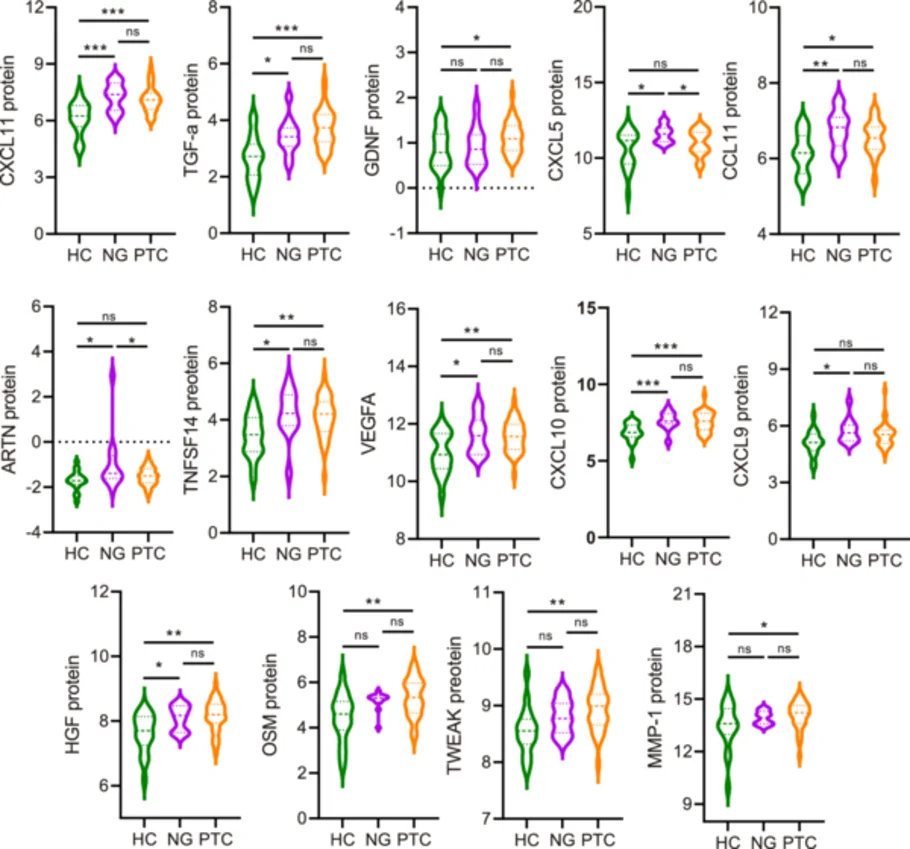
The protein expression of CXCL11, TGF‐α, GDNF, CXCL5, ARTN, TNFSF14, CCL11, VEGFA, CXCL10, CXCL9, HGF, OSM, TWEAK, and MMP‐1 were displayed. The expression levels of inflammatory proteins were examined using the proximity extension assay detection method. The protein expression was displayed through scatter plots in the healthy control group (HC, *n* = 18), nodular goiter group (NG, *n* = 12), and papillary thyroid carcinoma (PTC, *n* = 34). *p < 0.05; **p < 0.01; ***p < 0.001.

The expression levels of proteins included in the optimal diagnostic scheme (include logistic regression and LASSO algorithm), namely CXCL11, CCL11, TGF‐α, GDNF, CXCL5, ARTN, TNFSF14, CXCL9, CXCL10, HGF, OSM, TWEAK, MMP‐1, and CXCL5 as well as proteins associated with common risk factors, namely TNFSF14, CCL11, VEGFA, TGF‐α, CXCL11, and CXCL10, were presented in Figure 4. Comparatively, nodular goiter exhibited significant elevations in the proteins CXCL11, TGF‐α, CXCL5, ARTN, TNFSF14, CCL11, VEGFA, CXCL10, CXCL9, and HGF, compared to the healthy control group. Similarly, PTC demonstrated significant elevations in the proteins CXCL11, TGF‐α, GDNF, TNFSF14, CCL11, VEGFA, CXCL10, OSM, TWEAK, and MMP‐1. Additionally, CXCL5 and ARTN proteins experienced notable decreases in the PTC group compared to the nodular goiter group.

## 3 DISCUSSION

This study utilized PEA technology to analyze the expression of 96 inflammatory proteins across three groups. Furthermore, we investigated the diagnostic potential of differentially expressed proteins among these groups. Logistic regression results showed that TGF‐α + CXCL11 exhibited promising diagnostic capabilities in distinguishing nodular goiter from healthy control (AUC = 0.88) and in distinguishing PTC from healthy control (AUC = 0.89), and GDNF + CXCL5 + ARTN (AUC = 0.87) demonstrated optimal diagnostic effectiveness in distinguishing PTC from nodular goiter. LASSO algorithm results showed that TGF‐α + CXCL11 + TNFSF14 + CCL11 + CXCL9 + ARTN (AUC = 0.92) exhibited promising diagnostic capabilities in distinguishing nodular goiter from healthy control; TGF‐α + CXCL11 + CXCL10 + HGF + OSM (AUC = 0.93) showed potential as diagnostic biomarkers for distinguishing PTC from healthy control; and TGF‐α + TWEAK + CCL11 + GDNF + CXCL9 + MMP‐1 + CXCL5 + ARTN (AUC = 0.93) demonstrated optimal diagnostic effectiveness in distinguishing PTC from nodular goiter. Finally, this study revealed that six proteins, namely TNFSF14, CCL11, VEGFA, TGF‐α, CXCL11, and CXCL10, are common risk factors for the occurrence of both nodular goiter and PTC when compared to the healthy control group.

With advancements in medical imaging techniques, the detection rate of thyroid nodules has significantly increased among the most prevalent types of thyroid nodules, namely PTC and nodular goiter. Surgical excision is commonly employed for treating PTC, whereas many cases of nodular goiter do not necessitate surgery. Radiofrequency ablation and laser ablation are generally utilized as the primary methods for thermally ablating nodular goiter. The issue of inconclusive preoperative diagnosis of thyroid nodules and excessive diagnosis of benign nodules has become increasingly prominent. Unnecessary surgeries have amplified the burden on patients and their families while diminishing the patient's quality of life. Fine‐needle aspiration biopsy of the thyroid is considered the gold standard for preoperative diagnosis; nevertheless, it is an invasive procedure that heightens the physical and mental stress on patients, as well as the economic burden. Hence, there is an urgent need to discover a noninvasive and more accurate method for preoperative diagnosis. Previous research has indicated that proteins may function as potential diagnostic biomarkers for distinguishing between PTC, nodular goiter, and healthy control. Preoperative serum markers thyroglobulin and 1‐25‐dihydroxyvitamin D have been proposed as potential tools for discriminating thyroid cancer [9, 10]. Serum levels of insulin‐like growth factor 1 in thyroid cancer patients were significantly higher compared to those in healthy control and patients with benign thyroid nodules, highlighting its potential as a diagnostic marker for distinguishing PTC from healthy control (AUC = 0.71) [11]. When preoperatively combined with measurements of thyroid‐stimulating hormone, systemic immune‐inflammation index, lymphocyte–monocyte ratio, and ultrasound characteristics, it is possible to differentiate between PTC and benign thyroid nodules accurately (AUC = 0.808) [12]. Moreover, the combined diagnosis of ISG15 and PLXNB2 can distinguish PTC from healthy control and even stage I PTC from healthy control [13]. However, there is a lack of systematic research on the diagnostic role of inflammatory markers in PTC. In biliary tract cancer, PEA results show that the combination of IL‐6 and IL‐15 is the strongest predictor of survival [14]. The CEA + IL6 combined diagnosis can serve as a biomarker for early diagnosis of colorectal cancer [15]. In this study, the combination of logistic regression and the LASSO algorithm both exhibit good diagnostic performance; however, the combination derived from the LASSO algorithm outperforms logistic regression, albeit with a higher number of protein features.

Chemokines, a class of small cell‐signaling proteins secreted, play a crucial role in the inflammation‐mediated network associated with cancer, contributing to the maintenance and development of tumor‐related inflammation [16]. Chemokines and their receptors are instrumental in shaping the immune microenvironment in thyroid cancer [17]. This study identifies CXCL11, CXCL5, CXCL10, CXCL9, and CCL11 as potential diagnostic markers or risk factors for PTC. Previous research has indicated heightened expression of CXCL11 in PTC patients, associated with promoting endothelial cell migration and angiogenesis [18]. In addition, the level of CCL11 was significantly elevated in advanced PTC patients [19]. Building upon prior findings, this study reinforces the association of heightened CXCL11 and CCL11 expression with PTC and their role as risk factors for its occurrence. Furthermore, the upregulation of CXCL5 in TC cells and tissues has been observed [20]. However, this study found that although CXCL5 expression was upregulated, there was no significant upregulation between the PTC and healthy control groups. In addition, CXCL10 is downregulated in PTC and linked to a lower overall survival rate in thyroid cancer patients [21, 22]. In contrast to previous studies, this research suggests that CXCL10 is highly expressed in PTC and serves as a risk factor for its occurrence. It requires further analysis with an expanded sample size to elucidate the reasons for this discrepancy.

Moreover, the study implicates VEGFA and TGF‐α as risk factors for PTC occurrence. TGF‐α was overexpressed in PTC, and inhibiting TGF‐α has been shown to enhance the sensitivity of TC cells to cisplatin and inhibit tumor growth [23]. VEGF‐A promotes tumor angiogenesis and growth in PTC [24, 25]. Studies have demonstrated the role of TGF‐α in promoting VEGF‐A expression in cancer, thereby stimulating tumor angiogenesis. Consistent with past research, this study reaffirms the heightened expression of VEGFA and TGF‐α in PTC, underlining their significance as risk factors for its occurrence.

In addition, this study found that GDNF, ARTN, HGF, TWEAK, OSM, and MMP‐1 can become diagnostic markers for PTC. GDNF activates GFRα1/RET, promotes the proliferation of thyroid cancer cells, and exerts the role of oncogenes [26, 27]. The ARTN protein, a subtype of the glial cell line‐derived neurotrophic factor (GDNF) family ligands (GFL), is the fourth member of the GDNF family. Recent studies have indicated that the ARTN protein plays a significant role in promoting cell chemotaxis, adhesion, and migration, thus holding crucial implications in mediating tumor cell invasion and metastasis [28]. ARTN is overexpressed in cervical cancer tissue, which promotes the proliferation, invasion, and migration of cervical cancer cells [29]. HGF and MMP‐1 expression was obviously increased in PTC and promoted PTC development [30, 31]. This study demonstrates that levels of GDNF and ARTN are increased in PTC patients compared to those with nodular goiter, particularly with a significant increase in ARTN levels. In addition, HGF, TWEAK, OSM, and MMP‐1 are increased in PTC patients compared to those in healthy control, while those had no obvious change between PTC and nodular goiter patients.

The present research has several limitations. First, the study's small sample size necessitates future expansion to validate the results. Second, the analysis relies on PEA results and requires validation using ELISA or other protein detection techniques. Furthermore, variations in age and gender among the collected specimens may introduce bias into the results. In addition, this study collected samples from only one hospital, which may affect the reliability of the results. Therefore, in subsequent experiments, we will collect external hospital samples for validation. Next, there is no relevant reagent kit for testing the combined diagnostic proteins in clinical practice; if the Olink Target 96 inflammation panel is directly used for testing, the cost will be relatively expensive (2500 RMB).

## 4 CONCLUSION

This study offers potential biomarkers for distinguishing between PTC and nodular goiter in clinical practice, thereby aiding in developing treatment strategies and mitigating overtreatment. Furthermore, compared to logistic regression, the results from the LASSO algorithm demonstrate better diagnostic efficacy, albeit requiring more protein combinations (Table S9). This implies that while the LASSO algorithm yields superior diagnostic outcomes, it also entails higher costs. Therefore, further research is needed to strike a balance between diagnostic accuracy and healthcare expenditure.

## 5 METHODS

### Patients selection

This study was focused on individuals with nodular goiter and PTC at the Department of Thyroid Surgery in our hospital between January 2023 and June 2023. The PTC patients were assessed and categorized based on histopathological results using the criteria outlined by the World Health Organization. Specifically, patients with PTC had postoperative histopathological diagnoses confirming the presence of PTC, while those with nodular goiter were diagnosed postoperatively through pathological results. Healthy volunteers who participated in the study underwent comprehensive physical examinations at our hospital and were confirmed to be free of thyroid‐related diseases following thyroid ultrasound examinations. Additionally, all participants provided informed consent before inclusion in the study. Exclusion criteria for the study participants were: (1) Presence of systemic diseases involving hypertension, diabetes, cardiovascular, gastrointestinal, renal, or pulmonary diseases, or other cancer. (2) Recurrent PTC. (3) Presence of autoimmune thyroid diseases. (4) History of surgery within 6 months before the study. (5) Smoking. (6) Use of thyroid‐related medications before testing. These criteria were put in place to ensure that the study focused on a specific cohort and minimized potential confounding factors.

### Samples collection and storage

The study included 18 healthy volunteers (healthy control), 12 patients with nodular goiter, and 34 patients with PTC. A blood sample of 5 mL was collected from each participant and then subjected to centrifugation to obtain the serum. The serum samples were stored at −80°C for further analysis.

### PEA testing

To assess inflammatory proteins, the PEA analysis was performed using the Olink Target 96 inflammation panel (Olink Bioscience). The experimental procedures followed the instructions provided with the Olink Target 96 inflammation panel. Briefly, proteins extracted from 20 μL serum were immunologically linked with the oligonucleotide sequences provided in the Olink Target 96 inflammation panel. Then, the paired oligonucleotide sequences were amplified using DNA polymerase. The amplified sequences were measured by qPCR detection (Signature Q100; Olink), and the data of qPCR detection were represented as normalized protein expression (NPX) using NPX Signature Software (Olink). The definition of NPX can be found in a previous study [32].

### Statistical analysis

Data analysis was conducted with SPSS version 19.0. Continuous variables were expressed as the mean ± standard deviation and analyzed via one‐way ANOVA. Count data were presented as percentages and analyzed using Fisher's exact test. Gene Ontology terms and Kyoto Encyclopedia of Genes and Genomes (KEGG) pathway enrichment analysis was analyzed in the online website (https://www.xiantaozi.com/products), with an FDR threshold of <0.05. The protein network was analyzed by the STRING database. Receiver operating characteristic (ROC) curve analysis assessed the AUC, cutoff value, sensitivity, and specificity. The diagnostic effectiveness improves as the AUC increases. In addition, a combined diagnostic marker for thyroid cancer was developed using LASSO algorithm (https://www.xiantaozi.com/products). The correlation between the different inflammatory proteins was evaluated by Spearman's correlation coefficients. Finally, a binary logistic regression model was applied to analyze risk factors, with the inflammatory proteins serving as independent variables while accounting for age and sex as confounding factors.

## AUTHOR CONTRIBUTIONS

**Yongqin Pan**: Conceptualization; methodology; statistical analysis; data curation; writing—original manuscript. **Mingxi Xu**: Conceptualization; statistical analysis; revision. **Tsz Hong Chong**: Investigation. **Siping Xie**: Investigation. **Kunsong Huang**: Investigation. **Guanghao Wang**: Investigation. **Yuhua Ma**: Investigation. **Jinyi Li**: Conceptualization; statistical analysis; revision. **Wah Yang**: Conceptualization; methodology; statistical analysis; data curation; writing—original manuscript. All authors have read and agreed to the published version of the manuscript.

## CONFLICT OF INTEREST STATEMENT

The authors declare no conflict of interest.

## ETHICS STATEMENT

The study was approved by IRB of the First Affiliated Hospital of Jinan University (KY‐2022‐114) and conducted in accordance with the Declaration of Helsinki.

## Supporting information


**Table S1:** The baseline data of healthy control, nodular goiter, and PTC.
**Table S2:** The diagnostic accuracy of inflammatory protein in distinguishing healthy control, nodular goiter, and PTC.
**Table S3:** The diagnostic accuracy of CXCL11, CXCL10, and CCL11 in distinguishing PTC from healthy control.
**Table S4:** The diagnostic accuracy of CXCL11, CXCL10, and CCL11 in distinguishing PTC from nodular goiter.
**Table S5:** The diagnostic accuracy of TGF‐α, CXCL11, and CXCL10 in distinguishing nodular goiter from healthy control.
**Table S6:** The diagnostic accuracy of TGF‐α, CXCL11, and CXCL10 in distinguishing PTC from nodular goiters.
**Table S7:** The diagnostic accuracy of GDNF, MMP‐1, CXCL5, and ARTN in distinguishing nodular goiters from healthy control.
**Table S8:** The diagnostic accuracy of GDNF, MMP‐1, CXCL5, and ARTN in distinguishing PTC from healthy control.
**Table S9:** The diagnostic accuracy in distinguishing healthy control, nodular goiters, and PTC by LASSO algorithm and logistic regression.

## Data Availability

This article does not generate new sequencing data and scripts. Supporting Information (tables, graphical abstract, slides, videos, Chinese translated version, and update materials) may be found in the online DOI or iMeta Science http://www.imeta.science/imetaomics/.
