# Supplementary material for: Combined detection of inflammatory proteins is beneficial for diagnosing the papillary thyroid carcinoma and nodular goiter
Source: IMetaOmics. 2024 Jul 2;1(1):e14. doi: 10.1002/imo2.14 (PMC12806474; doi:10.1002/imo2.14)
Supplement: Supplementary file 1 — Table S1: The baseline data of healthy control, nodular goiter, and PTC. Table S2: The diagnostic accuracy of inflammatory protein in distinguishing healthy control, nodular goiter, and PTC. Table S3: The diagnostic accuracy of CXCL11, CXCL10, and CCL11 in distinguishing PTC from healthy control. Table S4: The diagnostic accuracy of CXCL11, CXCL10, and CCL11 in distinguishing PTC from nodular goiter. Table S5: The diagnostic accuracy of TGF‐α, CXCL11, and CXCL10 in distinguishing nodular goiter from healthy control. Table S6: The diagnostic accuracy of TGF‐α, CXCL11, and CXCL10 in distinguishing PTC from nodular goiters. Table S7: The diagnostic accuracy of GDNF, MMP‐1, CXCL5, and ARTN in distinguishing nodular goiters from healthy control. Table S8: The diagnostic accuracy of GDNF, MMP‐1, CXCL5, and ARTN in distinguishing PTC from healthy control. Table S9: The diagnostic accuracy in distinguishing healthy control, nodular goiters, and PTC by LASSO algorithm and logistic regression. [file IMO2-1-e14-s001.docx]

**Supplementary Table 1** The baseline data of healthy control, nodular goiter, and PTC

| Baseline data | Healthy control  (n=18) | Nodular goiter  (n=12) | PTC  (n=34) | P |
| --- | --- | --- | --- | --- |
| Gender (male) | 12 (66.67%) | 2 (20%) | 15 (44.12%) | 0.028 |
| Age | 32.44±8.431 | 46.75±11.23 | 37.47±12.45 | 0.005 |
| BMI | 24.07±4.903 | 22.70±3.159 | 23.71±4.007 | 0.852 |
| Uric acid | 351.78±65.924 | 356.07±100.240 | 376.33±102.959 | 0.642 |
| Total cholesterol | 5.14±1.771 | 6.14±1.032 | 5.32±1.212 | 0.276 |
| Triglycerides | 1.18±0.938 | 2.45±2.903 | 1.50±1.659 | 0.272 |
| High-density lipoprotein | 1.24±0.195 | 1.39±0.355 | 1.28±0.294 | 0.512 |
| Low-density lipoprotein | 3.12±0.962 | 3.48±0.790 | 3.08±0.965 | 0.591 |
| Free triiodothyronine | — | 4.86±0.525 | 5.31±0.484 | 0.013 |
| Free thyroxine | — | 9.08±2.257 | 11.25±1.668 | 0.001 |
| Thyrotropin | — | 1.55±1.056 | 1.75±0.994 | 0.593 |
| Thyroid globulin antibody | — | 0.00 (0.00, 0.31) | 0.08 (0.00, 0.66) | 0.480 |
| Thyroid peroxidase antibody | — | 0.51 (0.36,54.15) | 0.72 (0.35, 29.06) | 0.994 |
| Intact parathyroid hormone | — | 29.44±13.159 | 37.67±14.071 | 0.257 |
| Carcinoembryonic antigen | — | 1.69±0.901 | 1.37±0.912 | 0.502 |

**Supplementary Table 2** The diagnostic accuracy of inflammatory protein in distinguishing healthy control, nodular goiter, and PTC

| Inflammatory protein | nodular goiter vs healthy control | PTC vs healthy control | PTC vs nodular goiter |
| --- | --- | --- | --- |
| TGF-alpha | 0.769 | **0.848** | 0.635 |
| CXCL11 | **0.838** | **0.812** | 0.583 |
| CXCL10 | **0.824** | **0.781** | 0.532 |
| MCP4 | 0.694 | 0.76 | 0.554 |
| CCL28 | 0.708 | 0.778 | 0.581 |
| VEGFA | 0.745 | 0.742 | 0.505 |
| HGF | 0.75 | 0.76 | 0.564 |
| DNER | 0.713 | 0.765 | 0.596 |
| TWEAK | 0.699 | 0.773 | 0.627 |
| LAP-TGF-beta1 | 0.671 | 0.708 | 0.566 |
| OSM | 0.731 | 0.752 | 0.559 |
| CCL4 | 0.755 | 0.747 | 0.564 |
| MCP_1 | 0.69 | 0.717 | 0.502 |
| TNFSF14 | 0.778 | 0.748 | 0.551 |
| IL7 | 0.639 | 0.678 | 0.551 |
| CCL3 | 0.708 | 0.703 | 0.551 |
| AXIN1 | 0.681 | 0.699 | 0.502 |
| CCL11 | **0.815** | 0.709 | 0.623 |
| Beta-NGF | 0.671 | 0.678 | 0.517 |
| MCP2 | 0.644 | 0.706 | 0.566 |
| CX3CL1 | 0.694 | 0.722 | 0.532 |
| IL12B | 0.704 | 0.699 | 0.505 |
| IFN-gamma | 0.727 | 0.719 | 0.502 |
| IL2 | 0.667 | 0.691 | 0.578 |
| GDNF | 0.532 | 0.68 | **0.657** |
| CD40 | 0.727 | 0.681 | 0.551 |
| IL6 | 0.708 | 0.716 | 0.512 |
| CXCL9 | 0.778 | 0.678 | 0.613 |
| IL8 | 0.644 | 0.641 | 0.5 |
| MMP1 | 0.606 | 0.665 | **0.645** |
| CXCL6 | 0.62 | 0.639 | 0.532 |
| TNF | 0.583 | 0.637 | 0.554 |
| LIF | 0.657 | 0.675 | 0.527 |
| CCL19 | 0.546 | 0.645 | 0.598 |
| CXCL5 | 0.718 | 0.606 | **0.699** |
| ARTN | 0.759 | 0.623 | **0.642** |

**Supplementary Table 3** The diagnostic accuracy of CXCL11, CXCL10, and CCL11 in distinguishing PTC from healthy control

| Variables | Cut-off value | Area under the curve | Confidence interval | Sensitivity | Specificity |
| --- | --- | --- | --- | --- | --- |
| CXCL11 | ＞6.8231 | 0.81 | 0.686-0.938 | 0.71 | 0.83 |
| CXCL10 | ＞7.6828 | 0.78 | 0.656-0.906 | 0.47 | 1.00 |
| CCL11 | ＞6.2113 | 0.71 | 0.551-0.867 | 0.82 | 0.61 |
| C1 | ＞10.463 | 0.83 | 0.704-0.946 | 0.79 | 0.78 |
| C2 | ＞9.5768 | 0.79 | 0.655-0.917 | 0.85 | 0.61 |
| C3 | ＞12.836 | 0.78 | 0.656-0.906 | 0.50 | 0.94 |
| C4 | ＞23.907 | 0.82 | 0.697-0.934 | 0.88 | 0.61 |

**Note:** C1: CXCL11+CXCL10; C2: CXCL11+ CCL11; C3: CXCL10+ CCL11; C4: CXCL11+CXCL10+ CCL11.

**Supplementary Table 4** The diagnostic accuracy of CXCL11, CXCL10, and CCL11 in distinguishing PTC from nodular goiter

| Variables | Cut-off value | Area under the curve | Confidence interval | Sensitivity | Specificity |
| --- | --- | --- | --- | --- | --- |
| CXCL11 | ＜7.4862 | 0.58 | 0.375-0.791 | 0.79 | 0.50 |
| CXCL10 | ＜7.6393 | 0.47 | 0.281-0.655 | 0.50 | 0.58 |
| CCL11 | ＜6.9 | 0.62 | 0.420-0.825 | 0.82 | 0.50 |
| C1 | ＜11.554 | 0.56 | 0.347-0.771 | 0.76 | 0.50 |
| C2 | ＜10.483 | 0.58 | 0.377-0.790 | 0.68 | 0.58 |
| C3 | ＜12.674 | 0.59 | 0.401-0.786 | 0.47 | 0.75 |
| C4 | ＜26.307 | 0.57 | 0.369-0.773 | 0.76 | 0.50 |

**Note:** C1: CXCL11+CXCL10; C2: CXCL11+ CCL11; C3: CXCL10+ CCL11; C4: CXCL11+CXCL10+ CCL11.

**Supplementary Table 5** The diagnostic accuracy of TGF-α, CXCL11, and CXCL10 in distinguishing nodular goiter from healthy control

| Variables | Cut-off value | Area under the curve | Confidence interval | Sensitivity | Specificity |
| --- | --- | --- | --- | --- | --- |
| TGF-alpha | ＞3.0009 | 0.77 | 0.593 - 0.944 | 0.83 | 0.72 |
| CXCL11 | ＞7.1794 | 0.84 | 0.690 - 0.986 | 0.67 | 0.94 |
| CXCL10 | ＞7.3432 | 0.82 | 0.661 - 0.987 | 0.83 | 0.78 |
| C1 | ＞8.9526 | 0.88 | 0.760 - 1.000 | 0.75 | 0.89 |
| C2 | ＞9.0312 | 0.86 | 0.717 - 0.996 | 0.83 | 0.78 |
| C3 | ＞11.526 | 0.86 | 0.722 - 1.000 | 0.67 | 0.94 |
| C4 | ＞10.273 | 0.88 | 0.746 - 1.000 | 0.75 | 0.89 |

**Note:** C1: TGF-α+CXCL11; C2: TGF-α+CXCL10; C3: CXCL11+CXCL10; C4: TGF-α+CXCL11+CXCL10.

**Supplementary Table 6** The diagnostic accuracy of TGF-α, CXCL11, and CXCL10 in distinguishing PTC from nodular goiters

| Variables | Cut-off value | Area under the curve | Confidence interval | Sensitivity | Specificity |
| --- | --- | --- | --- | --- | --- |
| TGF-alpha | ＞3.7385 | 0.64 | 0.453 - 0.817 | 0.50 | 0.83 |
| CXCL11 | ＜7.4862 | 0.58 | 0.375 - 0.791 | 0.79 | 0.50 |
| CXCL10 | ＞7.6393 | 0.47 | 0.281 - 0.655 | 0.50 | 0.58 |
| C1 | ＜9.3412 | 0.47 | 0.284 - 0.662 | 0.56 | 0.58 |
| C2 | ＞10.355 | 0.57 | 0.385 - 0.762 | 0.32 | 0.92 |
| C3 | ＜11.957 | 0.55 | 0.342 - 0.766 | 0.76 | 0.50 |
| C4 | ＞11.608 | 0.51 | 0.311 - 0.704 | 0.24 | 0.92 |

**Note:** C1: TGF-α+CXCL11; C2: TGF-α+CXCL10; C3: CXCL11+CXCL10; C4: TGF-α+CXCL11+CXCL10.

**Supplementary Table 7** The diagnostic accuracy of GDNF, MMP-1, CXCL5, and ARTN in distinguishing nodular goiters from healthy control

| Variables | Cut-off value | Area under the curve | Confidence interval | Sensitivity | Specificity |
| --- | --- | --- | --- | --- | --- |
| GDNF | ＞0.40824 | 0.53 | 0.318 - 0.747 | 1.00 | 0.17 |
| MMP-1 | ＞13.357 | 0.61 | 0.397 - 0.816 | 1.00 | 0.39 |
| CXCL5 | ＞10.471 | 0.72 | 0.526 - 0.909 | 1.00 | 0.44 |
| ARTN | ＞-1.6316 | 0.76 | 0.578 - 0.940 | 0.92 | 0.61 |
| C2 | ＞10.081 | 0.72 | 0.532 - 0.903 | 0.67 | 0.72 |
| C7 | ＜-5.3018 | 0.72 | 0.531 - 0.904 | 0.83 | 0.61 |
| C8 | ＜-3.4459 | 0.84 | 0.692 - 0.984 | 0.75 | 0.83 |
| C9 | ＜4.4279 | 0.67 | 0.465 - 0.868 | 1.00 | 0.39 |
| C10 | ＞2.2386 | 0.76 | 0.592 - 0.936 | 0.92 | 0.61 |
| C11 | ＜-1.9643 | 0.81 | 0.655 - 0.965 | 0.75 | 0.78 |

**Note:** C2: GDNF+CXCL5; C7: GDNF+MMP-1+CXCL5; C8: GDNF+MMP-1+ARTN; C9: GDNF+CXCL5+ARTN; C10: MMP-1+CXCL5+ARTN; C11: GDNF+MMP-1+CXCL5+ ARTN.

**Supplementary Table 8** The diagnostic accuracy of GDNF, MMP-1, CXCL5, and ARTN in distinguishing PTC from healthy control

| Variables | Cut-off value | Area under the curve | Confidence interval | Sensitivity | Specificity |
| --- | --- | --- | --- | --- | --- |
| GDNF | ＞0.72945 | 0.68 | 0.511 - 0.849 | 0.88 | 0.50 |
| MMP-1 | ＞13.605 | 0.67 | 0.498 - 0.832 | 0.79 | 0.56 |
| CXCL5 | ＜11.001 | 0.39 | 0.221 - 0.566 | 0.50 | 0.56 |
| ARTN | ＞-1.565 | 0.62 | 0.459 - 0.786 | 0.59 | 0.72 |
| C2 | ＜10.306 | 0.49 | 0.304 - 0.679 | 0.88 | 0.28 |
| C7 | ＞-5.7587 | 0.50 | 0.317 - 0.686 | 0.94 | 0.22 |
| C8 | ＞-3.3007 | 0.50 | 0.315 - 0.685 | 0.76 | 0.44 |
| C9 | ＞4.0641 | 0.63 | 0.454 - 0.805 | 0.88 | 0.50 |
| C10 | ＞0.81612 | 0.57 | 0.388 - 0.746 | 0.91 | 0.33 |
| C11 | ＞-1.8431 | 0.53 | 0.340 - 0.709 | 0.76 | 0.44 |

**Note:** C2: GDNF+CXCL5; C7: GDNF+MMP-1+CXCL5; C8: GDNF+MMP-1+ARTN; C9: GDNF+CXCL5+ARTN; C10: MMP-1+CXCL5+ARTN; C11: GDNF+MMP-1+CXCL5+ ARTN.

**Supplementary Table 9** The diagnostic accuracy in distinguishing healthy control, nodular goiters, and PTC by LASSO algorithm and logistic regression.

| Variables | Analysis | Proteins combination | Area under the curve | Sensitivity | Specificity |
| --- | --- | --- | --- | --- | --- |
| nodular goiters vs healthy control | logistic regression | 2^a^ | 0.86 | 0.67 | 0.94 |
|  | LASSO algorithm | 6^b^ | 0.92 | 0.83 | 0.94 |
| PTC vs healthy control | logistic regression | 2^a^ | 0.89 | 0.76 | 0.89 |
|  | LASSO algorithm | 5^c^ | 0.93 | 0.88 | 0.89 |
| PTC vs nodular goiters | logistic regression | 3^d^ | 0.87 | 0.74 | 0.92 |
|  | LASSO algorithm | 8^e^ | 0.93 | 0.76 | 1.00 |

^a^TGF-α+CXCL11;

^b^TGF-alpha+CXCL11+TNFSF14+CCL11+CXCL9+ARTN;

^c^TGF-alpha+CXCL11+CXCL10+HGF+OSM;

^d^GDNF+ CXCL5+ARTN;

^e^TGF-alpha+TWEAK+CCL11+GDNF+CXCL9+MMP-1+CXCL5+ARTN
